# Schizophrenia outcomes in the 21st century: A systematic review

**DOI:** 10.1002/brb3.2172

**Published:** 2021-05-15

**Authors:** Peter Huxley, Anne Krayer, Rob Poole, Louise Prendergast, Sanjaya Aryal, Richard Warner

**Affiliations:** ^1^ Centre for Mental Health and Society School of Health Sciences Bangor University Bangor UK; ^2^ Department of Sociology University of Essex Colchester UK; ^3^ Clinical Professor of Psychiatry and Adjunct Professor of Anthropology University of Colorado Denver CO USA

**Keywords:** annualized remission rate, employment, recovery, social outcome

## Abstract

**Objective:**

We report a review of outcomes in schizophrenia in the twenty‐first century, replicating and extending work undertaken by the late Richard Warner in his seminal book, “*Recovery from Schizophrenia: Psychiatry and Political Economy”* (1985;2004).

**Method:**

Warner's methods were followed as closely as possible. Only observational/naturalistic studies were included. Six scientific databases were searched from 2000 to 2020. 6,640 records were retrieved. 47 met inclusion criteria.

**Results:**

Overall, complete recovery is higher in this study than in Warner's (37.75% cf 20.4%), especially for first episode psychosis (FEP) (57.1% cf 20.7%). Clinical recovery, annualized remission rate (ARR), and employment outcomes were significantly superior for first episode psychosis compared with multiple episode psychosis (MEP). ARR shows a trend toward reduction over time, from 2.2 before the financial crash of 2008 to 1.6 after (t = 1.85 *df* 40 *p* = .07). The decline is statistically significant for the MEP group (t = 2.32 *df*18 *p* = .03). There were no differences in outcome by region, sample characteristics, outcome measures used, or quality of studies. Heterogeneity of clinical outcome measures across the literature makes evidence synthesis difficult. Weak and inconsistent reporting of functional and employment outcomes mean that findings lack meaning with respect to lived experience.

**Conclusion:**

Future research strategies should aim to reduce heterogeneity in clinical outcome measures and to increase the emphasis on capture and reporting of more sophisticated measures of social and functional outcome. Outcome domains should be disaggregated rather than conflated into unitary recovery constructs.

## Graphical abstract

A systematic review of the 21st century outcomes in schizophrenia.

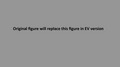

## 1 INTRODUCTION

This paper reports a review of outcomes in schizophrenia in the twenty‐first century and is an extension of the work undertaken by the late Dr Richard Warner in his seminal book, “Recovery from Schizophrenia: Psychiatry and Political Economy” (1985 (Warner, 1985); 2004 (Warner, 2004)). The present work was started with Dr Warner's involvement, and the preliminary results were presented at the XVII World Congress of Psychiatry in Berlin in 2017. Here, we present the final results based on research findings published between 2000 and 2020. Initially, we intended to conduct a systematic review and meta‐analysis, but study heterogeneity and paucity of data, including data capture problems and reporting weaknesses, means that meta‐analysis was not possible. The relevance of these issues is considered further below.

## 2 BACKGROUND

In 1985, Warner used empirical evidence to strongly challenge the prevailing view of schizophrenia, which largely arose through the influence of Kraepelin (Kendler, 2020), who suggested that psychosis was strongly characterized by poor clinical and social outcomes. Since then, evidence from epidemiological, sociological, psychological, and biological studies has made many aspects of the Kraepelinian model of schizophrenia unsustainable (Murray, 2017). Few hold to the concept of schizophrenia as a unitary disorder, or even, as Bleuler suggested when he coined the term, a distinct group of psychoses. (Bleuler and Zinkin, 1950)

Richard Warner was one of the first to systematically bring together and analyze data from outcome studies of schizophrenia in the twentieth century. Although we (and others) regularly refer to his work in the 2004 edition of Recovery from Schizophrenia, it is important to remember that the first edition was published in 1985, almost two decades earlier. He was interested in shedding light on whether “schizophrenia is an inherently catastrophic illness from which only modern psychiatric treatment can afford relief; or that it is a condition with a considerable spontaneous recovery rate upon which treatment has little long‐term effect.” (p.60). Most importantly, he showed that conditions such as social and political attitudes and the state of the economy, “mould the course and outcome of the illness and influence, along with other factors, its incidence.” (p. xii) and by implication, outcome. Subsequent work has confirmed his challenge to the Kraepelinian notion of a dementia‐like psychotic process, mainly determined by biological factors. His work has contemporary relevance.

Warner distinguished between “complete recovery” and “social recovery.” He defined the former as loss of psychiatric symptoms and return to preillness level of functioning, where as he defined social recovery in functional terms, economic and residential independence with low social disruption, an important component of which is employment.

Since Warner's 1985 edition of Recovery from Schizophrenia, there has been intense debate over the concept of recovery. In particular, there has been controversy over the ownership of definitions of recovery; the preferred model of patient self‐definition of recovery creates methodological problems for quantitative researchers, whereas the emergence of a “Recovery Model” in statutory services has led to accusations that service definitions of “recovery” are sometimes euphemisms for withholding care and treatment. Consequently, the literature is marked by a variety of definitions of “recovery,” “complete recovery,” and “partial recovery,” some of which are agreed by some groups of researchers, some of which are idiosyncratic (Cornish, 2020; Liberman & Kopelowicz, 2002; Livingston, 2020). We must acknowledge the existence of these differences and their impact on our conclusions.

Warner (Warner, 2009) provided a neat attempt to weave the scientific and experiential approaches to “recovery” together. He rightly pointed out that “the proportion of patients considered to have recovered will depend on how rigorously recovery is defined” (p61). A number of issues of definition are of significance here. First, it seems to us that to combine both clinical remission and social function into a single recovery definition is not helpful and risks the loss of important outcome information. As Warner himself said, measures of social functioning are hard to standardize and can cover a wide range of behaviors and activities. In the experiential approach to recovery, individuals value different behaviors and activities (work, family contact etc) differently and the importance attributed to them may vary overtime. Warner hypothesized that social environment had a profound effect on the outcome of psychosis, so that circumstances that support people to social inclusion led to a virtuous cycle of improved well‐being. This being the case, his thesis was that the political economy is a key modifiable factor in improving rates of recovery. In line with this, we believe that employment status ought to receive more attention as an outcome indicator. This is not without its difficulties, which we mention later. Nevertheless, we have included employment outcomes in this review.

Second, with regard to the rigor of the definition, it is the case that the length of “recovery” needs to be defined. Warner himself did not include an explicit time criterion in his definition, although a 12 month criterion was implicit in his selection of studies. Although the Remission17 criteria for proposed evidence‐based and consensus‐based criteria for defining clinical remission does include a 6‐month time criterion, this is not adhered to in all studies.

We believe that the most satisfactory definition of “complete recovery” is clinical remission and sustained functional outcomes, which should include employment, for at least 6 months, but it is in the nature of a review of this sort that the relevant information is not always available in the public domain.

In his most recent review, Warner included 114 studies from the 1,880 to 2004. He found that recovery rates overall were little changed since the 1900s. In the last period of his review between 1976 and 1995, he separately reported on clinical recovery and social recovery for people with first episodes of psychosis (FEP) and for those who experienced multiple episodes of psychosis (MEP). In MEP, the mean complete recovery rate was 20% and social recovery 33%, which was not very different from the overall recovery rate from 1901 to 1910 (20% and 41%, respectively). In FEP, recovery occurred in 27%, and social recovery in 35%; higher compared to the earliest figure he gave (for the years 1921–1940) which were 12% and 28%, respectively. He was unable to present detailed findings for what he termed “the developing world” (that is, low‐ and middle‐income countries or LMICs), but he did break down the US and UK results. From 1976 to 1995, complete recovery occurred in 17% in the USA and 19% in the UK, while social recovery in the USA was 43% and in the UK 30%.

Since Warner's work there have been several reviews, some of which report pooled outcomes (Cohen et al., 2008; Leucht & Lasser, 2006; Van Eck et al., 2018), and two reviews of reviews (Miettunen, 2015; NeuRA (Neurosciences Research Australia). Remission and recovery), 2020). The reviews reporting pooled outcome data use very different methodologies. In fact, the heterogeneity that scholars bemoan in individual outcome studies is equally present in the reviews (cited chronologically in the Supplementary Material A). For instance, Menezes (Menezes et al., 2006) did not require included studies to report both clinical remission and social outcome and did not specify a time period for outcomes. Clemmensen et al. (Clemmensen et al., 2012) looked at FEP and included patients with mood and other disorders (mixed samples) as well as some retrospective studies, hospital discharge and outpatient samples. The studies were categorized as reporting outcome by use of both the General Functioning Scale (GFS) and study‐specific functioning (SSF) outcomes. The GFS studies were categorized by the study authors as a “poor” outcome (score ≤50), “moderate” outcome (score 51–70), or “good” outcome (score >70), but there was no consistency in the use of these precise cutoff points across all studies. The authors subjectively and independently rated the SSF outcome data in the papers as “poor,” “moderate,” or “good.”

In 2003, the Remission in Schizophrenia Working Group (RSWG) (Andreasen et al., 2005) proposed evidence‐based and consensus‐based criteria for defining clinical remission. Remission was defined as “a level of core symptoms (positive, negative, and disorganized) that does not interfere with an individual's behavior and is also below that required for an initial diagnosis of schizophrenia to be made according to the Diagnostic and Statistical Manual of Mental Disorder, fourth edition (DSM‐IV)” (Nasrallah & Lasser, 2006). AlAqeel and Margoleses’ review (AlAqeel & Margolese, 2012) used the RSWG criteria and included only those papers that provided data with a minimum six‐month follow‐up of patients—the length of follow‐up originally suggested by the RSWG.

Jääskeläinen et al. (Jääskeläinen et al., 2013) included both clinical and social outcomes using the RSWG definition of recovery with persistence for two years. They commented on the “high” heterogeneity of recovery estimates (I^2^ statistic=99.8%) and found a median annual recovery rate of 1.4%, with no statistically significant difference in outcome by gender. There was a significantly higher rate of recovery LMICs, as suggested by Warner and others (although this has been disputed by some (Cohen et al., 2008) and rejoindered by others (Bromet, 2008; Jablensky & Sartorius, 2008)). Their recovery figure for the 1976–1995 period (9.9%) was much lower than Warner's. They reported, however, that the strictness of the definition of recovery used had no effect on outcome results. The difference between their results and Warner's is almost certainly due to their use of a persistence criterion in the definition. We will examine the relevance of a persistence criterion in the analysis section of this paper.

Lally et al. (Lally et al., 2017) included FEP studies only and used Jääskeläinen's criteria for recovery but also examined improvement persisting over one year. Studies that failed to meet the Jääskelänen criteria were designated “broad criteria.” The pooled rate of clinical remission for all included diagnoses was 58% (56% for schizophrenia). Only 23% achieved full recovery. They reported no difference in remission rates by study quality, duration of follow‐up, study setting, or use of narrow/broad remission criteria/the RSWG criteria. Recovery rates were higher in Africa (73%; 2 studies only), Asia (66%; 2 studies only), and North America (65%; 17 studies) compared with Europe and Australia. In the most recent period, 2005–2016, recovery rates remained higher but not significantly so. Miettunen (Miettunen, 2015) reviewed systematic reviews of schizophrenia outcomes and reported an overall recovery rate of 13.5% and also found higher rates of recovery in poorer countries. NeuRA (NeuRA (Neurosciences Research Australia). Remission and recovery, 2020) reported a review of six reviews conducted through three search engines (all these engines are included in our searches). They suggest that the quality of the evidence in the six reviews is at best moderate, that the overall recovery rate for schizophrenia in the 21st century has been between 13% and 16%, and that the five year outcome for first episode is 58% clinical recovery, but they do not provide pooled averages for social or employment outcomes.

We do not believe that the existence of this marked heterogeneity should be a reason to cease all comparative outcome research. In our opinion, researchers should continue to strive to reduce heterogeneity and to use indicators where greater consensus can be achieved. Employment status is one such candidate. A contextualized measure of financial strain might be another. The advantage of the present review is not that it reduces heterogeneity but that it allows a meaningful longitudinal view because, by using the same methods as Warner, it compares like with like.

Since the first edition of Warner's book, there has been a substantial increase in outcome research: in first episode psychosis (FEP), in early onset, in intervention samples, and, most recently, in high‐risk groups. This has led to intense interest in the role of duration of untreated psychosis. This variable is excluded from the present review (as there is nothing in Warner's 1985 & 2004 editions to compare it with). Since Warner's original work there has been a growing awareness of the need to incorporate other features of recovery other than simply clinical remission (Andreasen et al., 2005; Emsley et al., 2011; Gorwood & Peuskens, 2012; Harvey, 2009; Karow et al., 2012; Lally et al., 2017; Lambert et al., 2009; Vita & Barlati, 2018). Employment is considered as an outcome in some reviews, but pooled data are not given (Cohen et al., 2008). None of the reviews (summarized in the Supplementary Material A) include employment status as an outcome indicator in spite of its growing relevance and evident support for its consideration. (Bouwmans et al., 2015; Kinoshita et al., 2010; Lloyd‐Evans et al., 2013; Srinivasan & Thara, 1997; Srinivasan & Thara, 1999; Tsang et al., 2010).

As indicated earlier, wherever the data were presented, we have included employment outcome in this review.

The purpose of the present review is to assess the extent to which Warner's conclusions, and the conclusions of subsequent reviews, hold in the twenty‐first century. Are remission rates stable, are they influenced by different definitions of remission and by different persistence criteria, are they affected by duration of follow‐up or other study features? How do clinical, social, and employment outcomes differ in MEP and FEP studies and are outcomes better in LMICs? To explore the evidence supporting Warner's hypothesis concerning the importance of changes in the political economy, we have taken the opportunity to look for any noticeable difference in outcomes for data gathered before and after the 2008 crash.

## 3 METHOD

We followed Warner's methods as closely as possible. As in his original review, only observational/naturalistic studies were included, and study samples comprised at least 80% individuals with diagnoses of schizophrenia, schizophreniform, and schizoaffective disorder (i.e., broadly defined “schizophrenia”) with at least 6 months follow‐up (Warner included those of one year or over, but we adhered to the more recent RSWG criteria). In studies where a “schizophrenia” subsample of 30 or more cases was fully described independently within the paper, we used only those data. In addition, if early intervention or first episode studies included persons with a schizophrenia diagnosis and reported these results separately, then the schizophrenia group results are also included in our analysis. FEP is defined as patients who are making their first treatment contact for psychotic symptoms OR are in their first episode of psychosis AND do not meet diagnostic criteria for an affective disorder (i.e., only schizophrenia‐spectrum diagnoses included).

Exclusion criteria were as follows:

- Age <18 years old at study inception (but not for FEP studies where no lower age limit was applied).
- source not written in English language;
- clinical trials;
- primary diagnosis other than schizophrenia (e.g., bipolar disorder);
- selected outpatient and hospital discharge samples;
- retrospective studies;
- cross‐sectional studies;
- small studies (*n* < 25);
- cognitive and neurological function only assessed;
- data gathered entirely or mainly in the 20th century.

We also excluded studies where outcome ratio data could not be computed (see also Hegarty (Hegarty et al., 1994)). Where a study was reported in more than one paper using the same data, the paper with usable and latest results was included (as in Jääskeläinen et al. (Jääskeläinen et al., 2013)). In some cases where different outcomes (clinical and functional) from the same study were reported in separate papers (e.g., Addington et al. (Addington et al., 2003; Addington et al., 2003)), both outcomes were included in the results, but the total number of subjects was adjusted to avoid double counting. Where a research group reported single study results separately for MEP and FEP cases, we entered both sets of results into the analysis, but did not double count respondents. We examined four types of outcome: clinical; social; complete recovery (which we defined as meeting both RSWP and Warner criteria), and employment (measures of social recovery are listed in Supplementary Material B). Given Warner's thesis regarding the influence of the political economy, we felt it was important to consider social and employment outcomes (employed/not) as well as clinical outcome, and complete recovery (as defined above). In some studies, the only usable outcome data were on employment (Segarra et al., 2012). The abrupt contextual changes in the global economy in 2008 (the generally accepted date of the global financial crash) created an opportunity to assess any changes from pre‐ to postcrisis.

RW and PH began the search and review process, using Warner's inclusion and exclusion criteria and made decisions jointly whether to include or exclude studies. At this stage (2015–6), more than 700 papers were under review. Warner's untimely death occurred before the process could be completed. Searches were undertaken again in 2017–18 and updated in 2020.

•*Search terms*: terms schizo* OR psychos*s OR psychotic AND recovery OR outcome* OR remission OR longitudinal OR course OR follow‐up in a title search.
•Period: 1 Jan 2000‐30 June 2020
•Databases:
  •Science Direct
  •Proquest (Social Sciences Collection)
  •PsycArticles
  •Cinahl (Ebscohost)
  •Medline (Ebscohost)
  •Web of Science (Biosis, Core, Scielo)

​

Decisions on study inclusion were undertaken in pairs between PH, AK, SA, and LP. In all cases where it was possible, the decisions were confirmed by reference to Warner's own notes shared with PH in 2016. In the event of disagreement, a third opinion was sought from one of the other authors of this paper.

To explore any impact of the financial crash, we divided the studies into those where data collection was entirely completed before 2008, and those whose data were collected entirely after 2008. Remaining studies where data collection included 2008 were assigned to the period in which the majority of data were gathered.

We also divided the studies depending upon their definition of outcome and recovery. We contrasted studies using the RSWG definition of clinical remission (Andreasen et al., 2005) and those that did not. The location of the study was examined categorizing data collection areas into Europe, North America, and the rest of the world (a catch‐all necessitated by low numbers of studies). We further compared outcomes in first episode psychosis (FEP) or early intervention study samples with all outcomes in non‐FEP/Early Intervention studies, labeled various or multiple episodes of psychosis (MEP) (called “mixed duration” by Warner). Length of follow‐up was divided into 6 months (our minimum) or longer than 6 months. Where follow‐up was repeated at more than one time point, the final assessment was used. This enabled us to include the longest available outcomes while avoiding double counting.

### 3.1 Statistical procedures

Recovery estimates are presented as pooled averages or as medians. For change over time, we used the same year categories as Warner. For economic comparisons, we used the per capita income statistics as recommended by Cohen et al. (Cohen et al., 2008) based on the latest figures provided by the World Bank (World Bank). Analysis by regions compared studies conducted in the USA, Europe, and the rest of the world. For comparisons by definition of recovery, we used the Andreasen et al. criteria (2005) (Andreasen et al., 2005) (RSWG) versus studies using other definitions. Warner did not include an explicit persistence criterion in his definition of recovery but others have done, and so we examine all the outcomes by the persistence criterion used in the included studies, using analysis of variance. In relation to study quality, we followed the MOOSE criteria for meta‐analysis in observational studies (Stroup et al., 2000) and consulted subsequent relevant guidance (Aromataris and Munn, 2020; Briggs, 2017; Deeks et al., 2008; Huedo‐Medina et al., 2006).

Variable distributions were checked. Skewed variables were transformed appropriately, for example, the social/functional outcome variable. Annualized recovery rate was calculated by dividing the remission rate by the length of follow‐up (see Jääskeläinen et al. 2013) (Jääskeläinen et al., 2013). Means of the independent continuous outcome variables were analyzed in relation to the dependent variables using *t* tests or one‐way ANOVA, and relationships between continuous variables by correlational analysis. Heterogeneity was tested using the I^2^ statistic.

## 4 RESULTS

**FIGURE 1:**
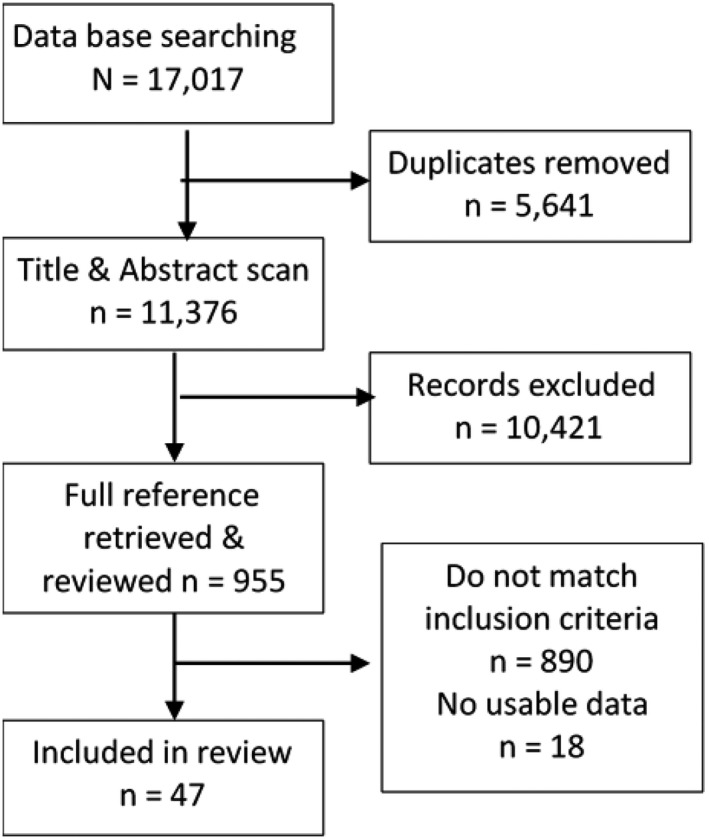
Search strategy

A total of 47 studies (Addington et al., 2012; Addington & Addington, 2008; Addington, Leriger, et al., 2003; Addington, Young, et al., 2003; Alem et al., 2009; Arceo & Ulloa,^2019^; Bachmann et al., 2007; Bodén et al., 2014; Carter et al., 2011; Češková et al.,^2007^, ^2011^; Chan et al., 2015, 2019; Chang et al., 2014; Chua et al., 2019; Economou et al., 2011; Giraud‐Baro et al., 2016; Hassan & Taha, 2011; Heering et al., 2015; Hegelstad et al., 2012; Jaracz et al., 2015; Johansson et al., 2018; Johnson et al., 2012; Kebede et al., 2005; Kurihara et al., 2011; Lauronen et al., 2007; Malla et al., 2002, 2006; Mattsson et al., 2008; Norman et al., 2014, 2018; Revier et al., 2015; Ritsner et al., 2014; Ruggeri et al., 2004; Saravanan et al., 2010; Schennach et al., 2020; Segarra et al., 2012; Shibre et al., 2015; Singh et al., 2007; Spellmann et al., 2012; Strålin et al., October 2018; Suresh et al., 2012; Torgalsbøen et al., 2014; Üçok et al., 2011; Verdoux et al., 2010; Verma et al., 2012; Whitty et al., 2008; Wolter et al., 2010) (full details are given in Supplementary Material B) met the inclusion criteria and provided data for year of study, definition of outcome, stage of illness, length of follow‐up (in all but one instance follow‐up was 12 months or more), and region (Figure 1).

Some of these gave only functional or employment outcomes. In three instances, the study reported separate data for two outcomes. Accordingly, 50 data sets were entered into the analysis. The number of studies that followed the Warner criteria for clinical remission was 43. The clinical results below are based on these 43 studies unless otherwise specified. In some other studies, data were presented in a format that could not be interpreted for our present purposes. Such studies usually involved the use of predictive regression models, and raw outcome data were not reported. In all instances, we attempted to, but were unable to, access the raw data. Examples of the types of studies excluded are given in the Supplementary Material C.

The total number of (unduplicated) participants is 13,430 (FEP: 6,049; MEP: 7,381). Clinical outcome was reported in 43 papers (11,048 participants), functional or social outcomes in 20 papers (5,602 participants), and employment outcomes in 23 papers (9,990 participants). Since we are interested in changes in the recent past, our outcomes are reported in table 1 alongside Warner's (2004) results from the latter part of the 20th century, that is, 1945–2000 (derived from Warner 2004; Table 3.1 pages 64–76).

**TABLE 1:** Complete and social recovery: comparison of 20th and 21st century studies

	Complete % (mean; sd)		Social % (mean; sd)	
	MEP*	FEP**	MEP	FEP
Warner (1945–2000) *N* = 64	20.37 (11.3)	20.7 (11.52)	41.1 (16.6)	40.6 (17.7)
Huxley et al. (2000–2020) *N* = 43	37.75 (14.9)	57.14 (15.4)	43.5 (23.0)	47.3 (20.7)

Abbreviations: FEP, First episode psychosis; MEP, Multi Episode Psychosis.

* *p* <.05

** *p* <.001.

### 4.1 Study quality

There was no relationship between the quality rating and any of the outcome measures, including clinical outcomes (t = 1.379 *df* 41 *p* =.175; mean difference =8.817; SE diff=6.394 95% CI −4.095 to 21.730). The Jääskeläinen review (Jääskeläinen et al., 2013) found considerable heterogeneity in the recovery rate (I^2^ statistic =99.8%), and we too found high heterogeneity in the clinical outcome variable in our included studies (I^2^ statistic =97.1%).

### 4.2 Definition used

Most of the studies used the RSWG criteria, but a very few added the duration requirement, some at 6 months, (Heering et al., 2015; Kurihara et al., 2011) some one year, (Wolter et al., 2010) and others required two years. (Schennach et al., 2020) A comparison between groups of studies that had no duration criterion (36%), or a criterion of six months (42%), or a criterion of twelve months or more (22%) showed no significant differences in any outcome. The outcomes for those studies that used RSWG criteria compared to those using “other” criteria also showed no differences. In all outcomes, the “other” definitions had better outcomes by a few percentage points, with the exception of ARR which was higher in the RSWG studies (ARR 2.2 cf 1.9) but was not significant.

### 4.3 Clinical and social outcomes and stage of illness

Table 1 shows the mean (pooled average) outcomes in the original Warner work (from post‐World War Two to the end of the century) together with the 21st century results from the present review. Complete recovery improves significantly in the MEP group, but social recovery is not significantly improved in either MEP or FEP. The most striking feature is the significantly higher complete recovery rate (57%) in FEP studies in the present review.

Warner presented the recovery data by decade, and Jääskeläinen et al. (Jääskeläinen et al., 2013) did the same. By their own account, the latter authors used a stricter definition of recovery. We reanalyzed Warner's data to obtain the median recovery rates of FEP and ME by decade, and these are presented in Table 2.

**TABLE 2:** Comparison of median (%) recovery rate by decades

Decades	Warner (FEP)	Warner (MEP)	Jääskeläinen et al. 2013
Pre 1941	18.5	29.0	13.0
1941–1955	24.5	31.5	17.7
1956–1975	21.0	19.0	16.9
1976–1995	25.5	12.0	9.9
After 1996	29.0	13.6	6.0

Note

Data derived from Warner 2004 (Warner, 2004); Table 3.1 pages 64–76

Our figures for the first two decades of the 21st century continue trend of improvement previously reported by Warner. Our median is 54.0%. This is consistent with the mean figures given in Table 1. Similarly, our median for MEP is also considerably improved at 33.45 and is a return to the median levels Warner observed between 1941 and 1955. Possible reasons for these changes are considered in the discussion.

### 4.4 Annualized recovery rate (ARR)

Using the ARR as defined by Jääskeläinen and colleagues(Jääskeläinen et al., 2013), (who found a median ARR of 1.4%) we found a median ARR of 2.2%. Warner's median ARR for the last period in his review (1980 to 2000) was 2.9. In our data, the ARR shows a trend toward significant reduction over time, reducing to 1.6 after the financial crash of 2008 from 2.2 before (t = 1.85 *df* 40 *p* =.07). The reduction is statistically significant for the MEP group (t = 2.32 *df*18 *p* =.03).

Table 3 compares all the outcomes for the MEP and FEP groups in our review. Because ARR and social outcome were both skewed, we used log‐transformed variables. Clinical remission, the annualized recovery rate, and employment are all significantly superior for the FEP group, but social outcome is not.

**TABLE 3:** Multiple episode compared to first episode outcomes

Outcome variable	t	*df*	sig	Mean difference	SE of difference	95% CI	
						Lower	Upper
Employment	−2.43	36	0.020*	−13.70	5.65	−25.16	−2.26
Clinical remission	−4.61	47	0.000**	−19.43	4.21	−27.90	−10.95
Social/functional (log)	−0.896	22	0.380	−0.79	0.08	−0.26	−0.11
Annualized recovery rate (log)	−2.63	46	0.001**	−0.31	0.12	−0.55	−0.07

* *p* <.05

** *p* <.001.

### 4.5 Location

While there is a trend for employment outcome rate to be better in the rest of the world (45% sd 19.9; 8 papers) than in Europe (38.6% sd 18.9; 19 papers) and North America (35.4% sd 24.6; 5 papers), there is no statistically significant relationship between any of the outcomes and region. There was no difference in the regional annualized recovery rate. This result holds for both FEP and MEP studies. Using RSWG studies only, there are still no significant differences by region.

Comparing the 5 LMIC countries with the HICs showed that only employment was significantly different (better in LMIC t = 2.18 *df* 30 *p* =.037).

### 4.6 Sample characteristics (Sample size, % male, % follow‐up, and length follow‐up)

There are no significant associations between these variable (sample size, gender distribution, percentage followed up, and length of follow‐up) and any outcome measure. The results are the same in both the MEP and FEP groups.

### 4.7 Measures used

PANSS was the most commonly used clinical outcome measure (62.5% of studies). GAF was the most commonly used functional outcome measure (39.3% of studies). There are no differences in clinical, social, employment, or ARR outcomes in either MEP or FEP cases when PANSS and GAF are used compared to the other measures used.

### 4.8 Year of data collection

In studies conducted after 2008, good clinical and employment outcomes both decline. Functional outcome improves, but the functional data are highly skewed. Although positive clinical outcome is reduced from a pooled average of 49% before 2008 to 45.6% after, this change is not significant. Employment outcome is markedly worse after the crash (employed 34.9%) than before (employed 42.3%) (as one would expect), but this is not statistically significant. These findings apply to both the FEP and MEP groups.

## 5 DISCUSSION

Our review has some limitations. The most significant of these is heterogeneity of methods and outcome criteria between studies. This is a limitation which is intrinsic to reviews of naturalistic outcome studies, and it has affected all of the previous reviews. It precludes overconfident conclusions or a claim of definitive findings, especially in those subanalyses where the number of studies is small. While most of our results are indicative only, they do shed light on the multifaceted nature of recovery and on important temporal trends.

This review of 21st century studies tends to confirm one of Warner's key assertions that a significant proportion of people who receive a schizophrenia diagnosis make a good recovery. There are some significant new findings. Generally speaking, these do not reach statistical significance owing to wide confidence intervals, but they resonate with many other findings on the impact of poverty, employment, and other social factors (Wolter et al., 2010).

While we have found that rates of complete recovery have increased substantially for people experiencing a first episode of psychosis in the 21st century, not all of our findings are positive. Findings by both Warner and Jääskeläinen showed decreasing annualized rates of recovery over time, and we have found a continuing decline in ARR since Warner's review. Differences in method and criteria almost certainly account for differences in their figures, particularly the use of a persistence criterion by Jääskeläinen. Nonetheless, the trend is the same in all three reviews.

People with multiple episodes fare much worse than people who respond well to intervention for FEP (Table 1). While it has long been recognized that relapse increases the risk of subsequent relapse, something appears to have changed. It is reasonable to speculate that this might be due to changed priorities in mental health policy since the end of the era of deinstitutionalization (roughly 1955–1995). High‐income countries (HICs) have made huge efforts to improve outcomes from FEPs. There has been substantial investment in specialist FEP/early intervention services, which contrasts starkly with disinvestment, loss of research interest and, some would say, neglect of rehabilitation and other services for people with MEP. (Poole et al., 2013).

There is an apparent paradox that an improved rate of complete recovery has been accompanied by a deteriorating ARR. The same clinical and research focus on FEP in the UK and USA might account for this. It is possible that better early intervention on first presentation of psychosis results in an increasingly high proportion of those who will achieve recovery experiencing this immediately, with a consequent reduction in recovery rates for those who relapse, who predominate in the annualized sample. A similar explanation is unrelated to treatment. Overtime, changes in social attitudes may mean that a history of a single episode of psychosis may have become less marginalizing, say with respect to employment, but that recurrent episodes, with attendant disruption to employment and housing, have a much more marginalizing effect, a poor social outcome then contributing to poor clinical outcome.

A further temporal change is a trend toward better outcomes prior to the 2008 crash, and for this to decline somewhat afterward. Both findings are compatible with Warner's emphasis on the political economy as a key factor influencing people's ability to recover. Warner notes (pp38‐40) that when the business cycle goes into recovery, patients in the low income groups disproportionately pay the psychological price of adapting to new jobs, in new locations, with new colleagues, all of which result in new illnesses or the exacerbation of existing ones.

There is a trend to better outcomes in the non‐UK non‐US studies, but this is hard to interpret. The category includes data from LMICs and some HICs, and the number is very small. The finding is of interest, but cannot be taken to show that recovery rates are better in LMICs. More importance can be attached to the fact that, overall, participants in the non‐UK non‐US group probably had poorer access to treatment. If treatment accounted for improved outcomes, you would expect the trend in this group to be in the opposite direction to one we have found.

The same caveats must attach to the finding of a trend toward better employment outcomes in non‐UK non‐US studies, but there may be an important factor relating to better but less formal employment opportunities in LMIC settings. Although we cannot say that outcomes in LMICs are better, our review lends no credence to the idea that LMIC outcomes are worse. Warner took the view that outcomes were better in the developing world, and our limited findings are congruent with other recent findings (Jääskeläinen et al., 2013; Killaspy & Priebe, 2020; Miettunen, 2015).

Our findings of changes in outcomes over time, with possible attenuation of improvements in HICs after 2008 and outcomes probably no worse in LMICs (possible better), tend to suggest that Warner was right and that social factors are key determinants of recovery. It may be argued that a definition of recovery that includes employment will inevitably become less common in hard times, but this misses the point. Recovery and context cannot be separated. As suggested above, sustained employment is a measure of recovery, but employment is also known to improve clinical outcomes.

There must be some caution about the impact of the 2008 crash. Doubtless, its effects took time to work through. The collapse of the Lehman Brothers Bank (the first sign of problems) was in 2006. Arguably, studies conducted shortly after 2008 were less affected by the crash than those reporting later. It is, of course, possible to make other assumptions and take other cutoff points and the data are available for those who wish to do that. Also, it can be argued that the period since 2008 is too short to reveal significant differences in all of the outcomes.

Comparison of our findings and Warner's original findings shows significant improvements in rates of recovery FEP, with more disappointing results for MEP, especially post‐2008. There appears to have been no real improvement in social outcomes for either FEP or MEP. Rates of recovery are lower when a length of recovery criterion is applied, but trends are unaffected. Taken with Warner's, Miettunen's (Miettunen, 2015) and Jääskeläinen's (Jääskeläinen et al., 2013) findings, there may be a consistent decline in annualized recovery rates decade by decade. The research synthesis literature has found no consistent increases in recovery when defined solely by changes in clinical symptoms. As the published data do not permit robust analysis of social or employment outcomes, there is a pressing need, noted by other authors, for improvements in the capture and reporting of clinical and social outcomes. A reduction in methodological heterogeneity of studies would be a major step forward, with adoption of standard definitions of functional recovery and social outcomes. Having said that, we were unable to show that the greater homogeneity produced by using the RSWG standard definition of recovery led to any differences in reported outcomes compared to other definitions.

Our understanding of functional outcomes would be improved if employment outcomes were disaggregated into meaningful categories of type, length, security of employment, and remuneration rates. This is important for the evaluation of social interventions and system‐wide service improvements. Warner would strongly approve of such a development. One could argue on the basis of this and other reviews that a more profitable way forward might be to think in terms of outcome profiles based on several functional and clinical measures rather than conflating them as many definitions of “recovery” do.

To conclude, there is growing recognition that “outcome” is most meaningfully understood in terms of social parameters. A new approach is needed that does not ignore the biological and psychological aspects of psychosis but does place both causation and intervention firmly in their social context. Psychosis is a disorder where onset, course, and outcomes are profoundly affected by social factors. Recovery can only meaningfully be understood as a social phenomenon.

## CONFLICT OF INTEREST

None declared

## SUMMATIONS

- During the 21st century, the trend of improvement in rates of recovery appears to have continued, irrespective of how recovery is defined.
- Outcomes for first episode psychosis appear to be far better than for multi‐episode psychosis, which may be due to improvements in intervention, social attitudes, both, or neither. The predominance of multi‐episode individuals in annualized recovery rate data may account for the paradoxical deterioration in this parameter.
- Changes in the political economy appear to have an immediate impact in slow improvements in recovery rates, emphasizing the central importance of social factors.

​

## LIMITATIONS

- The studies included are highly heterogenous with respect to definitions of recovery and reporting of outcome parameters. Measures of social recovery tend to be crude or omitted altogether.
- The degree of heterogeneity in the literature precludes meta‐analysis
- All studies included are naturalistic, which improves relevance to clinical practice, but makes interpretation of impact of specific factors more difficult.

​

### PEER REVIEW

The peer review history for this article is available at https://publons.com/publon/10.1002/brb3.2172.

## Supporting information

Supplementary Material A

Supplementary Material B

Supplementary Material C

## Data Availability

The authors confirm that the data supporting the findings of this study are available within the article [and/or] its supplementary materials.
