# Supplementary material for: Schizophrenia outcomes in the 21st century: A systematic review
Source: Brain Behav. 2021 May 15;11(6):e02172. doi: 10.1002/brb3.2172 (PMC8213926; doi:10.1002/brb3.2172)
Supplement: Supplementary file 1 — Supplementary Material A [file BRB3-11-e02172-s002.docx]

**Supplementary Material A**: Table of 21st century reviews of outcomes in Schizophrenia (where pooled averages have been given)

| Review (date) | Sample type reported  (ME = Mixed episodes; FEP = First episode) | Total Number of papers | Years covered | Patient numbers at follow-up (unduplicated) | Good Clinical outcome %  (pooled averages) | Good Social /functional outcome %  (pooled averages) | Recovery % |
| --- | --- | --- | --- | --- | --- | --- | --- |
| Warner (2004) | Both | 26  (15 FEP) | 1976-1995 | Total 2,634  1207 FEP  1427 ME |  | 33  (FEP 35) | 20  (F2EP 27) |
| Menezes et al (2006) | FEP | 37 | 1966-2003 | 4,100 | 42.2 | 41.7 |  |
| Clemmensen et al (2012) | FEP | 21 | 1980-2011 | 422 | 15.4 | 14.3 |  |
| AlAqeel and Margolese (2012)* | Both | 27  (12 FEP) | 2005-2011 | Total 8,897  FEP 2644  ME 6253 |  |  | FEP  (Range 17-78)  35.6  ME  (Range 16 to 62)  37 |
| Jääskeläinen et al (2013).* | ME | 50 | 1823 -2011 | 8,994 |  |  | 16.4**  (IQR 8.1-20.0) |
| Lally et al (2017)* | FEP | 79 | 1976 -2016 | 19,072 | 58 |  | 38  (1997-2016 32)  (1976-1996 45) |
| This study (2020) | Both | 43  (FEP 23) | 2000-2020 | 14214  ME 7379  FEP 6835 | Overall 48.1  ME 37.75  FEP 57.14 | Overall 45.2  ME 43.5  FEP 47.3 | 47.6*  (IQR 31.5- 61.8)* |

- *Only including studies that SWG definition used*

***median 13.5*
