# Supplementary material for: Schizophrenia outcomes in the 21st century: A systematic review
Source: Brain Behav. 2021 May 15;11(6):e02172. doi: 10.1002/brb3.2172 (PMC8213926; doi:10.1002/brb3.2172)
Supplement: Supplementary file 2 — Supplementary Material B [file BRB3-11-e02172-s001.docx]

**Supplementary Material C:** Examples of studies excluded (by reason)

Outpatients only

Rafrafi R, Zaghdoudi L, Mahbouli M, et al. [Social outcome of schizophrenics in Tunisia: a transversal study of 60 patients]. *L'encephale*. 2009 Jun;35(3):234-240. DOI: 10.1016/j.encep.2008.05.001.

Selected sample (controls)

Pillmann F, Marneros A. Longitudinal follow-up in acute and transient psychotic disorders and schizophrenia. *British Journal of Psychiatry* 2005; 187:286-287. DOI: 10.1192/bjp.187.3.286

Clinical trial data

Wunderink L, Sytema S, Nienhuis F J, et al. Clinical recovery in first-episode psychosis. *Schizophrenia Bulletin*, 2009:*35*(2); 362–369. [doi: 10.1093/schbul/sbn143](https://doi.org/10.1093/schbul/sbn143)

McFarlane WR, Levin B, Travis L, et al. Clinical and functional outcomes after 2 years in the early detection and intervention for the prevention of psychosis multisite effectiveness trial. *Schizophr Bull*. 2015;41(1):30-43. doi:10.1093/schbul/sbu108

No usable data

Amoretti S, Rosa AR, Cabrera B, et al. Relationship between cognitive reserve and functional outcome in first-episode psychoses: a mediation analysis. *European Neuropsychopharmacology*, 2019: 29, S448–S449. [doi /10.1016/ j.euroneuro.2019.09.635](https://doi.org/10.1016/j.euroneuro.2019.09.635)

Vernal DL, Boldsen SK, Lauritsen MB, et al. Long-term outcome of early-onset compared to adult-onset schizophrenia: A nationwide Danish register study. *Schizophrenia Research*, 2020; 220: 123–129. https://doi.org/10.1016/j.schres.2020.03.045

No usable data : predictive modelling

Friis S, Melle I, Johannessen JO, et al. Early predictors of ten-year course in first-episode psychosis. In *Psychiatric Services* 2016;67: 438–443. American Psychiatric Association. https://doi.org/10.1176/appi.ps.201400558

Van Eck RM, Burger TJ, Vellinga A, et al. The Relationship Between Clinical and Personal Recovery in Patients With Schizophrenia Spectrum Disorders: A Systematic Review and Meta-analysis. *Schizophrenia Bulletin*, 2018;44(3):631–642. <https://doi.org/10.1093/schbul/sbx088>

Inadequate level of follow-up data

Chee, K. Y., Muhammad Dain, N. A., Abdul Aziz, S., Syed Mokhtar, S. S., Mat Junus, M., Zam Zam, R., … Cheah, Y. C. (2012). Outcomes of patients with first-episode schizophrenia at one-year follow-up: Findings from the National Mental Health Registry in Malaysia. *Asia-Pacific Psychiatry*, *4*(1), 30–39. <https://doi.org/10.1111/j.1758-5872.2011.00166.x>

Clinical high risk group only

Salokangas, R. K. R., Nieman, D. H., Heinimaa, M., Svirskis, T., Luutonen, S., From, T., … Ruhrmann, S. (2013, February). Psychosocial outcome in patients at clinical high risk of psychosis: A prospective follow-up. *Social Psychiatry and Psychiatric Epidemiology*. <https://doi.org/10.1007/s00127-012-0545-2>

Age

Lange, S. M. M., Meesters, P. D., Stek, M. L., Wunderink, L., Penninx, B. W. J. H., & Rhebergen, D. (2019). Course and predictors of symptomatic remission in late-life schizophrenia: A 5-year follow-up study in a Dutch psychiatric catchment area. *Schizophrenia Research*, *209*, 179–184. <https://doi.org/10.1016/j.schres.2019.04.025>

Second paper from an included study

Morgan C, Lappin J, Heslin M, et al. Reappraising the long-term course and outcome of psychotic disorders: the AESOP-10 study [published correction appears in Psychol Med. 2014 Oct;44(13):2727]. *Psychol Med*. 2014;44(13):2713-2726. doi:10.1017/S0033291714000282 [included in Revier et al 2015].

Paper not in English

Górna K, Jaracz K, Jaracz J, et al. [Social functioning and quality of life in schizophrenia patients --relationship with symptomatic remission and duration of illness]. Psychiatria Polska. 2014 Mar-Apr;48(2):277-288. PMID: 25016765

Crosssectional

Karow A, Naber D, Lambert M, et al. Remission as perceived by people with schizophrenia, family members and psychiatrists. *European Psychiatry*, 2012: *27*(6):426–431. https://doi.org/10.1016/j.eurpsy.2011.01.013

Retrospective

Poon MYC, Siu AMH, Ming SY. Outcome analysis of occupational therapy programme for persons with early psychosis. *Work*, 2010;37(1): 65–70. https://doi.org/10.3233/WOR-2010-1057
